# From Descriptive to Functional Genomics of Leukemias Focusing on Genome Engineering Techniques

**DOI:** 10.3390/ijms221810065

**Published:** 2021-09-17

**Authors:** Beata Balla, Florin Tripon, Claudia Banescu

**Affiliations:** 1 Genetics Department, George Emil Palade University of Medicine, Pharmacy, Science and Technology of Târgu Mureș, Strada Gheorghe Marinescu 38, 540139 Târgu Mureș, Romania; beakardos@gmail.com (B.B.); claudia.banescu@gmail.com (C.B.); 2 Center for Advanced Medical and Pharmaceutical Research, Genetics Laboratory, George Emil Palade University of Medicine, Pharmacy, Science and Technology of Târgu Mureș, Strada Gheorghe Marinescu 38, 540139 Târgu Mureș, Romania; 3 Clinical and Emergency County Hospital of Târgu Mureș, Strada Gheorghe Marinescu 50, 540136 Târgu Mureș, Romania

**Keywords:** genome engineering, CRISPR, DNA, leukemia

## Abstract

Genome engineering makes the precise manipulation of DNA sequences possible in a cell. Therefore, it is essential for understanding gene function. Meganucleases were the start of genome engineering, and it continued with the discovery of Zinc finger nucleases (ZFNs), followed by Transcription activator-like effector nucleases (TALENs). They can generate double-strand breaks at a desired target site in the genome, and therefore can be used to knock in mutations or knock out genes in the same way. Years later, genome engineering was transformed by the discovery of clustered regularly interspaced short palindromic repeats (CRISPR). Implementation of CRISPR systems involves recognition guided by RNA and the precise cleaving of DNA molecules. This property proves its utility in epigenetics and genome engineering. CRISPR has been and is being continuously successfully used to model mutations in leukemic cell lines and control gene expression. Furthermore, it is used to identify targets and discover drugs for immune therapies. The descriptive and functional genomics of leukemias is discussed in this study, with an emphasis on genome engineering methods. The CRISPR/Cas9 system’s challenges, viewpoints, limits, and solutions are also explored.

## 1. Introduction

Genome engineering makes the precise manipulation of DNA sequences possible in a cell. Therefore, it is essential for understanding gene function [1]. Genome editing began in the 1970s with the discovery of restriction enzymes by Werner Arber. Restriction enzymes are capable of recognizing and cutting at specific DNA sequences. Genome editing is therefore possible by introducing new nucleotide sequences at these sites [2,3]. However, restriction enzymes can only cut at particular sites, and this consists of their limitation of use depending on where we want the DNA to be cut. However, they are still commonly used today for some applications, and this discovery allowed the rise in future genome engineering techniques [4].

The discovery of meganucleases (naturally occurring restriction enzymes that can recognize 12–40 bp DNA sequences) was the initial step towards genome editing, followed by the discovery of zinc finger nucleases (ZFN) in the 1980s [4]. ZFNs comprise a nuclease domain and a specific zinc finger DNA-binding domain composed of a 3-base pair site on DNA. Multiple ZFNs can be combined to recognize longer sequences of nucleotides, increasing specificity and making it “customizable” to a target of interest [5]. ZFNs also proved advantageous for genome editing in plants, opening genome engineering to new species. While ZFNs were well-intentioned, the design and execution process was incredibly time-consuming and challenging [6]. In order to cut a specific site in the genome, ZFNs are built as a pair that can recognize two sequences flanking the site, one on the reverse strand and the other on the forward strand [7]. Upon binding the ZFNs on either side of the site, the pair of FokI domains dimerize and cut the DNA at the site, generating a double-strand break (DSB) with 5′ overhangs. DSBs can be repaired using either (a) non-homologous end joining (NHEJ) during any phase of the cell cycle—however, this mechanism often results in erroneous repair—or (b) homology-directed repair (HDR) during G2 or late S phase when the other sister chromatid is available to serve as a repair template [5].

Many genes in human cells and various model organisms have been effectively modified using ZFN-mediated targeting, paving the way for the development and application of genome editing tools [8]. The development of a three-finger protein to block the production of the BCR/ABL human oncogene in 1994 was the first demonstration of ZFN-driven gene disruption [9]. The researchers then used a human lymphoblast cell line derived from patients with chronic myeloid leukemia (CML) and a custom-designed ZFN to deliver DSBs to specific sites of the telomeric region of the mixed-lineage leukemia (MLL) gene breakpoint cluster region, as well as to analyze chromosomal structural abnormalities in MLL leukemogenesis by repairing DSB errors [8,9,10].

In 2011, new gene-editing technology made its way to the scientific world, involving transcription activator-like effector nucleases (TALENs) [11]. TALENs are similar to ZFNs as they are composed of a nuclease fused to DNA-binding domain sequences. However, TALENs recognize single nucleotides rather than relying on 3-base pair sites like ZFNs [6]. Transcription activator-like effector (TALE) repeats, which can naturally occur, consist of 10–30 repeat tandem arrays that bind and identify longer DNA sequences [5,12]. Each repetition is 33 to 35 amino acids long, with two neighboring amino acids indicating which of the four DNA base pairs it is specialized for. As a result, the repeats and base pairs in the target DNA sequences have a one-to-one connection. TALENs and ZFNs can cause DSBs at a specific locus in the genome. As a result, they can be utilized in the same way to knock out genes or introduce mutations [5,13,14].

TALENs have quickly established themselves as a viable alternative to ZFNs for genome editing. Although it is based on a non-specific FokI nuclease domain that may be fused to a customized DNA-binding domain, it differs from ZFNs in that the DNA binding domain is made up of highly conserved repeats derived from TALEs (proteins secreted by *Xanthomonas* bacteria) [15,16].

In 2012, genome engineering was revolutionized by discovering Clustered Regularly Interspaced Short Palindromic Repeats (CRISPR). Teams led by Jennifer Doudna and Emmanuelle Charpentier described the biochemical mechanism of CRISPR, which was until then known to be associated with innate immunity in bacteria. In 2013, a team led by Feng Zhang described how to use this system to edit eukaryotic DNA. The Cas9 protein is able to cleave double-strand DNA, and itis an RNA-guided nuclease discovered in the type II CRISPR immunity system of *Streptococcus pyogenes*. The well-known CRISPR/Cas9 system is made up of three components; a single guide RNA (sgRNA) specific to a target DNA sequence; Cas protein having a DNA endonuclease activity; and a trans-activating CRISPR RNA (tracrRNA) to interact with Cas9. The gRNA (approximately 20 base pairs in length) binds to the target DNA site and directs the Cas9 protein [17,18,19].

CRISPR systems work by identifying and precisely cleaving DNA molecules via RNA-guided recognition. Therefore their utility in epigenetics and genome engineering is proved[20]. CRISPR/Cas9 systems are generally made up ofa locus in the genome called the CRISPR array. Such arrays consist of about 20–50 base-pair (bp) repeats. These repeats are preceded by a so-called “leader” sequence that is rich in AT pairs and separated by so-called “spacers” with similar lengths [20,21].

CRISPR systems were initially identified and studied in prokaryotes in the 1990s. These systemsprotect against phage and plasmid genome exogenous DNA infection and are present in 90% of archaea and 50% of bacteria [22,23]. Once the CRISPR/Cas9 system from *Streptococcus pyogenes* (type II) was discovered in 2012, it was found to be a remarkably effective and efficient tool in DNA editing of higher eukaryotes. This finding opened a new world of possibilities in genetics [24]. The CRISPR/Cas system functions in three stages: the adaptive, processing, and interference stage [25,26,27]. CRISPR/Cas9 technology was first described in the *Escherichia coli* genomes as short repeats interspaced with short sequences and were officially named in 2002 [28]. This technology was initially used in 2013 to target the human genome, and its applications have since increased tremendously in biomedical research [29,30,31,32,33,34]. While CRISPR and TALENs enable editing at single-nucleotide resolution, CRISPR has become a more attractive alternative since it is less time-intensive and cost-effective [35,36].

**Figure 1:**
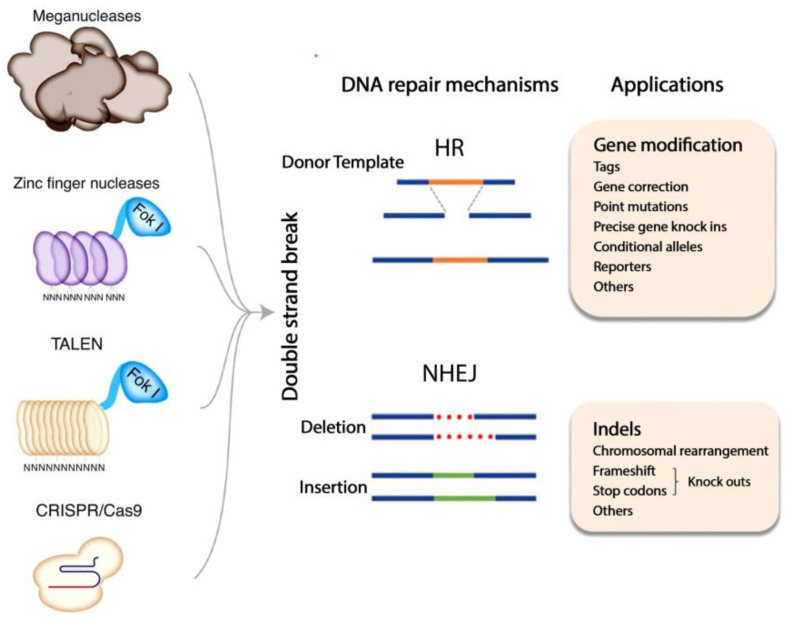
Summary of gene editing techniques. TALEN = transcription activator-like effector nucleases, CRISPR/Cas9 = clustered regularly interspaced short palindromic repeats- associated protein 9, HR = Homologous recombination, NHEJ = Non-homologous end joining [8,24,41,42,43].

In the last couple of years, CRISPR along with the Cas9 nucleases (CRISPR/Cas9), have revolutionized the possibilities for targeted genome editing [37]. These so-called RNA-guided endonucleases (RGENs) contain two RNA elements, CRISPR RNA (cRNA) on the one hand and its transactivating RNA (tracRNA) on the other hand. The two components can be fused, thus able to induce a targeted DSB [38]. When a corresponding DNA template is provided, specific gene sequences can be introduced into the DNA strand by homologous recombination (HR). However, the system’s efficiency is unclear in hematopoietic and progenitor cells, and it still remains a significant challenge [33,38,39,40]. The summary of gene-editing techniques is described in Figure 1 and characteristics of each technique are described in Table 1.

## 2. Gene Editing Techniques in Leukemia

Leukemias are generally classified as chronic or acute and as lymphocytic or myelogenous; the subtypes include chronic lymphocytic leukemia (CLL), chronic myeloid leukemia (CML), acute myeloid leukemia (AML), and acute lymphocytic leukemia (ALL).

### 2.1. Chronic Leukemia

CLLis a hematologic disorder in which the decision to treat is dependent on the presence or absence of symptoms. Some patients may require treatment upon diagnosis secondary to significant cytopenia or bulky lymphadenopathy, while others with stable or slowly progressive disease may simply be monitored closely [44].

CLL is one of the most common leukemias seen throughout the Western world. Despite the fact that chemotherapy works, the disease frequently returns, and resistance develops over time. Combining chemotherapy with monoclonal antibodies against specific surface markers, such as CD20, CD22, and CD23, appears to improve the outcome by giving a different strategy to induce apoptosis in clonal cells [45,46,47]. CLL cells also express the CD19 antigen on a constant basis, which is a suitable target for Chimeric Antigen Receptors (CARs) [45].

Unlike other B-cell lymphoproliferative disorders, the CLL genome lacks recurrent balanced chromosomal translocations. Therefore, it is relatively stable. Translocations that involve Immunoglobulin genes are relatively rare except *MYC*, *BCL2*, and *BCL3* rearrangements observed in about 2% of cases [48,49]. Studies of somatic copy number variations (CNV) using fluorescent in situ hybridization (FISH), single nucleotide polymorphism (SNP) analyses or karyotyping, identified recurrent lesions of prognostic relevance associated with CLL: most frequently del13q14 (in 50–60% of cases), del17p13 (in 5–10% of cases), del11q22 (in 6–20% of cases), trisomy 12 (in 10–18% of cases), and 2p gain (in 5–28% of cases) [50,51]. The minimally deleted regions point to putative CLL drivers: *MIR15A/MIR16-1* (*MIR15A/16-1*; microRNAs) and deleted in lymphocytic leukemia-2 (DLEU2) (long non-coding RNA, lncRNA) at 13q14, *TP53* at 17p13, and *ATM* and *BIRC3* at 11q22 [49]. The most frequent cytogenetic anomalies (deletions of chromosome 13q, 17p, 11q, and trisomy 12) were identified as markers with high prognostic relevance since they could stratify CLL patients into distinct groups according to the clinical progression of the disease and survival [51,52]. Chromosome 13q deletion has been described as an early event in CLL and is considered a favorable marker when isolated, whereas deletions of 11q and 17p and 2p gain are associated with progression and relapse [52]. Statistically significant associations between different cytogenetic abnormalities have been identified, as (i) 2p gain and 11q deletion, (ii) trisomy 12 with trisomy 18, and (iii) 14q deletion and translocations involving *BCL2*, *BCL3*, and *MYC*, but also with somatic mutations: (i) SF3B1 mutations and 11q deletion, (ii) *NOTCH1* mutations and trisomy 12, (iii) *MYD88* mutations and 13q deletion. The reason for such associations is unknown [49,53,54,55,56,57].

Deletion of a fragment on the long arm of chromosome 11 (11q22.3) associates a poor outcome and can be found in about 20% of CLL patients at diagnosis [56]. Although a variable size of the fragment can be deleted, the *ATM* gene is usually deleted in most cases. This gene has an essential role in the signaling and repair of DSB, and at diagnosis, approximately 10–20% of the CLL patients have at least one mutation. Simultaneous abnormalities have been identified in about 33% of CLL patients, consisting of heterozygous deletion of the specified region and different point mutations of the *ATM* gene. The result of these simultaneous mutations is the loss-of-function (LOF) of the protein. Consecutively the overall survival of CLL patients is significantly reduced [48,53,54,55,56,57,58,59].

*NOTCH1* is frequently mutated in CLL, but its functional impact on the disease remains unclear [60]. Using Mec-1 cell line models generated by CRISPR/Cas9 it has been shown that *NOTCH1* regulates homing and growth of the CLL cells by dictating the expression level of the *DUSP22* tumor suppressor gene [61]. By modulating a nuclear complex, *NOTCH1* changes the methylation of the *DUSP22* promoter in a specific way. This complex tunes up the activity of DNA methyltransferase 3A (*DNMT3A*). Moreover, these effects are amplified by PEST-domain mutations that prolong signaling and stabilize the molecule. CLL patients carrying a *NOTCH1*-mutated clone showed active chemotaxis to CCL19 chemokine and low levels of *DUSP22*. In addition, cells with *NOTCH1* mutation displayed a specific homing behavior xenograft model, localizing preferentially to the brain and spleen. These facts connect *DUSP22*, *NOTCH1*, and CCL19-driven chemotaxis in a single functional network, and suggest that by modulating the homing process, *NOTCH1* mutations may associate unfavorable prognosis in CLL patients [61,62].

The immunoglobulin heavy chain variable region (*IGHV*) mutations are generated during normal B-cell maturation through somatic hyper-mutation, which promotes immunoglobulin diversity [63]. Thus, B-cells that demonstrate mutated *IGHV* have completed somatic hypermutation, whereas B-cells lacking mutated *IGHV* have not undergone this process. It is thought that the development of CLL may emerge from B cells at varying stages of maturation. In 1999, it was first postulated that there was an association between CLL prognosis and *IGHV* mutation status [64,65,66].

CRISPR/Cas9 technique has been used to model common LOF alterations found in CLL (such as *BIRC3*, *TP53*, *CHD2*, *ATM*, *MGA*, *SAMHD1*) in the murine interleukin 3 (IL-3)-dependent pro-B cell line Ba/F3 [67,68]. For the possibility of multiplex gene editing, a quantitative polymerase chain reaction (qPCR) technique was developed in order to detect the identity and number of multiple sgRNAs in single cells. Genome aberrations induced by sgRNAs were analyzed in numerous individual cells using next-generation sequencing (NGS), PCR generation of targeted sequencing libraries, and droplet-based sequestration of single cells. The results demonstrate the possibility of single-cell DNA detection of gene edits for common LOFs generated by CRISPR/Cas9 gene editing. This method makes the assessment of a more significant number of cells (over 3000/sample) in multiple Cas9-target loci. Detection of single-cell modifications is essential for studying the possible effects of multiplex gene editing [67]. Hacken et al. (2020) demonstrated that co-transduction using six sgRNAs could generate several combinations of gene modifications. Therefore, the opportunity to study simultaneous mutations in a single cell with higher resolution is provided, thus making possible the functional genomic studies of disease drivers. Data support the use of multiplexed genome editing systems to study gene interactions in multiple diseases [67].

Ibrutinib is used for the treatment of CLL, but treatment response is variable in patients, even in patients with a similar genetic background. This variability in treatment response is thought to be related to differing CLL lineages and was described in a recent study characterizing CLL lineages by distinct DNA methylation and transcriptional profiles [69]. It is thought that this epigenetic reshuffling may further alter cell genomic neutrality while it is undergoing neoplastic transformation. Thus, alteration in epigenetic homeostasis may promote both oncogenic transformations as well as genetic alterations [19,69,70].

CD38 is a glycoprotein that serves as an ADP-ribosyl cyclase as well as a NAD glycohydrolase [71,72]. Its level of expression is linked to a bad prognosis in CLL patients, and it is exploited as a therapeutic target in multiple myeloma [18,73].

CML is a myeloproliferative cancer that is characterized by increased and uncontrolled growth of myeloid cells within the bone marrow and excessive accumulation of such cells in the blood [74,75]. CML is a blood cancer caused by *BCR/ABL1* fusion gene in a cell with the intrinsic or acquired biological ability to cause leukemia [76,77].

In recent years, a study focused on the use of genome-editing nucleases to disrupt *BCR/ABL1* as a treatment approach in CML. The CRISPR system has more therapeutic potential in CML patients, according to this study. In 2020, Chia-Hwa Lee and his colleagues disrupted *ABL1* in the human CML K562 cell line using a CRISPR/Cas9 lentiviral vector. They were able to show that disrupting *BCR/ABL1* resulted in a lower rate of proliferation [78].

### 2.2. Acute Leukemia

AML is the most common acute leukemia in adult patients and accounts for about 80% of acute leukemias in this population. It is a hematologic malignancy characterized by cellular hyperproliferation and impaired immature myeloid cell differentiation. These poorly differentiated cells infiltrate the bone marrow (BM), blood, and other tissues [79,80]. As the BM becomes filled with these cells, normal hematopoiesis is disrupted, resulting in characteristic findings of anemia, bleeding, and infection. AML may occur at any age but more commonly in adults and has an annual incidence rate of 3–4 cases per 100,000 [79,81,82,83,84].

AML models are usually caused by reciprocal chromosomal translocations. However, more than half of AML patients show a normal karyotype. Therefore, the majority of AML cases are driven by somatic mutations [38].

In over 60% of juvenile AML cases, reciprocal chromosomal translocations are the underlying genetic abnormality [85]. Rearrangements of the *MLL1/KMT2A* gene on chromosome 11q23 are the most common, accounting for about 25% of pediatric AML cases and 50% of neonatal AML cases [85,86].

Several somatic mutations, fusion-genes, and copy number aberrations (CNA) have been described for the AML genome. Some of them are recognized as independent biomarkers, others as candidate biomarkers for the patient’s prognosis, outcome, response to therapy, or overall survival. Briefly, we mention the impact of frequently identified somatic mutations, such as *FLT3*, *NPM1*, *TP53*, *RUNX1*, *ASXL1*, *IDH1*, *IDH2*, *DNMT3A*, *CEBPA*, *TET2*, *NRAS*, *KRAS*, *BCORL1* mutations [87,88,89,90,91,92]. In addition, several previously published papers detailed the descriptive genomics of AML patients and the well-known impact of the mentioned genetic abnormalities [93,94,95,96]. Due to the frequent co-occurrence of genetic abnormalities on the AML genome, it is essential to note the importance of the comprehensive genetic evaluation for an accurate diagnostic, prognostic, risk stratification, and therapy.

AML cells can express various stem cell and myeloid differentiation antigens on the cell membrane, such as CD33, CD34, CD123, CD135 [97].

The CRISPR/Cas9 system has been more frequently used for AML than the other types of leukemia. Therefore, we will focus on genome engineering techniques rather than descriptive genomics.

A powerful approach to in vivo identifying critical genes for leukemia cells is represented by forward genetic screens. For example, by in vivo RNA interference (RNAi) screens used in a murine AML model, many several leukemia-specific dependencies have been identified [98]. Even though RNAi screens are powerful techniques, they may result in a high rate of off-target effects. Therefore, RNAi screens have frequently been replaced by other techniques based on the CRISPR system in order to result in higher specificity and efficacy [98,99,100,101].

Studies applying genome sequencing have shown that malignancies in humans often carry mutations in more than four driver genes. This high level of genetic complexity, however, is difficult to recapitulate in mouse models when using conventional breeding. M. Jinek and his team in 2012 used the CRISPR/Cas9 system of genome editing in order to overcome this limitation [17]. Furthermore, by delivering combinations of Cas9 with a lentiviral vector and small guide RNAs (sgRNAs), researchers were able to modify five genes in one mouse hematopoietic stem cell (HSC), resulting in myeloid malignancy by clonal outgrowth [86]. They generated models of AML cells with coexisting mutations in genes encoding mediators of cytokine signaling, epigenetic modifiers, and transcription factors, therefore recapitulating the possible combinations of mutations seen in patients. Results suggested that sgRNA/Cas9 genome editing delivered by lentivirus could be used to engineer a broad spectrum of in vivo cancer models with the potential to better reflect the complexity of human disease [86].

Mutations induced to the 5′ exons of genes of interest have been targeted by some screening strategies using the CRISPR/Cas9 system. However, this approach can often lead to in-frame variants. In order to overcome this limitation, researchers targeted CRISPR/Cas9 mutagenesis in exons encoding for functional protein domains [102]. This can generate a higher frequency of null mutations, and the potency of negative selection can be substantially increased. Moreover, the magnitude of this natural selection can be used to identify the functional importance of studied protein domains. This approach can make it possible to identify protein domains that sustain cancer cells and could be suitable for drug targeting. For example, screening 192 chromatin regulatory domains in murine AML cells identified 19 dependencies and 6 known drug targets in a previously published study [102].

Research performed by Chen et al. in 2020 utilized a CRISPR-mediated pooled LOF screening method to investigate metabolic dependencies in AML [103]. It was found that pyridoxal kinase (PDXK) has an important role in AML. They also showed that in both in vivo and in vitro murine leukemia models, PDXK plays a crucial role in cell proliferation in AML. Knockdown of PDXK suppressed cell line proliferation derived from several murine AML models and human leukemic cell lines. It was also shown that *PDXK* depletion by single-guide RNA reduced in vivo Nras (G12D)/MLL/AF9 leukemic cell progression and prolonged overall survival in animals [103]. They also investigated the various mechanisms by which tumor growth is influenced by pyridoxal-5’-phosphate (PLP)-dependent metabolic enzymes. Out of 27 PLP-dependent enzymes, the CRISPR/Cas9 screen identified five that are critical for leukemic cell proliferation and expressed in AML cells [103]. To further determine the functional consequences of these enzymes, exogenous metabolites, including putrescine and aspartate, were added. These mimic the endogenous metabolites produced naturally byornithine decarboxylase 1 (ODC1) and glutamic-oxaloacetic transaminase 2 (GOT2), respectively. It was observed that the addition of these metabolites reduced the proliferative defect caused by the disruption of *PDXK*. Therefore, these results suggest that ODC1 and GOT2 are required for cell proliferation in AML and may have important treatment implications for future drug development targeting these two enzymes. In vivo studies involving PDXK, ODC1, and GOT2 knockout murine models should be considered to investigate mechanisms of action and further explore these enzymes as potential therapeutic targets. Additional research is needed to clarify if PDXK is functionally bound to ODC1/GOT2 in other non-leukemic cells [103,104].

Successful application of type II CRISPR/Cas9 system derived from Streptococcus pyogenes is able to transform genetic research in several different organisms [105]. Furthermore, given its flexibility and high efficiency, the system is appropriate for use in proof-of-principle studies. Genome-wide recessive genetic screens utilizing cancer cell lines demonstrate this technology’s potential to identify genes critical for cancer cell survival [106]. It is known that AML cells generally have a low mutational burden, and the mutation status of the *TP53* tumor suppressor gene is vital in AML prognosis. Therefore, it is essential to pursue screening involving AML lines in which the genetic background, namely *TP53* status, is well-defined [29,102,106,107,108,109].

CRISPR/Cas9 system has been employed to model cases of human clonal hematopoiesis with an indeterminate potential (CHIP) as well as AML [110]. Hematologic disorders with multiple mutated genes have been edited, including those encoding epigenetic regulators, transcriptional regulators, and signaling components in murine hematopoietic stem and progenitor cells [111]. Sequencing indels resulting from CRISPR/Cas9 tracked the clonal dynamics and demonstrated clonal expansion propagated by these leukemia-promoting cells and ultimately resulted in the development of AML in some of the recipient mice. Therefore, CRISPR/Cas9-induced multiplex mutagenesis may be utilized to design a variety of murine models emulating human hematological malignancies with complex genetic architectures [111].

Research investigating the relationship between AML and metabolic aberrancies has been performed using a CRISPR/Cas9 “drop out” screen applied as the gRNA library using metabolic genes that are strongly expressed in AML cells [108]. An aggregate of 2752 genes encoding transporters and metabolic enzymes were studied, identifying 236 genes noted to be abundantly expressed in leukemic cells [103]. A focused CRISPR/Cas9 sgRNA library targeting these 236 genes was introduced into Nras (G12D)/MLL/AF9 leukemic cells. This was combined with other sgRNAs associated with leukemogenesis and neutral sgRNAs of Renilla luciferase. The signal strength of each sgRNA from a continuous culture on days 1 to 9 was measured by deep sequencing. The control sgRNAs associated with genes known to be crucial for AML cell proliferation were depleted(e.g., *MYC*, *BCL2*, *MCL1*, *PCNA*, and *RPA1*), while sgRNAs targeting tumor suppressor *TP53* was enriched, and quantities of neutral sgRNAs remained essentially unchanged [103,112].

A CRISPR/Cas9 screen has also been used to identify critical markers of AML stem cells in vivo. This was done by targeting cell surface genes in a syngeneic MLL/AF9AML murine model and demonstrating that C-X-C chemokine receptor type 4 (CXCR4) was critical in the regulation for AML cell growth and survival. When the CXCR4 gene was deleted in AML cells, there was a disappearance of in vivo leukemic cells without impairment to their natural migration to the bone marrow. On the other hand, the CXCR4 ligand C-X-C motif chemokine 12 (CXCL12) was not found to be critical in the development of leukemia in recipient mice [100]. Evaluation of mutated *CXCR4* variant expression demonstrated that CXCR4 signaling is crucial for leukemic cells. The loss of CXCR4 signaling in vivo results in oxidative stress and differentiation. In conclusion, CXCR4 signaling is essential for AML stem cells by shielding them from differentiation regardless of CXCL12 stimulation [100].

AML cells divide quickly and have a wide range of metabolic abnormalities. As a result, medications targeting important enzymes in cancer cell metabolic reprogramming have been discovered, and inhibitors of isocitrate dehydrogenase (IDH) 1 and 2 (ivosidenib and enasidenib, respectively) have been approved by the FDA for the treatment of AML [38,104,113,114].

Due to AML’s complex sub-clonal heterogeneity, focusing on a single mutated gene triggers development of resistance mechanisms. Leukemic cells have an impressive array of adaptive capacities allowing them to escape the therapeutic attempts through targeted therapies [94].

Genome-wide CRISPR/Cas9 screening is used to identify targets for AML therapy. Initial screening using AML cell lines followed by an in vivo screen is performed. Results identified the mRNA decapping enzyme scavenger (*DCPS*) gene as playing a crucial role in AML cell survival [108]. The DCPS enzyme was shown to interact with components of pre-messenger RNA (pre-mRNA) metabolic pathways and spliceosomes by mass spectrometry. RG3039, a DCPS inhibitor that was initially developed to treat spinal muscular atrophy, demonstrated anti-leukemic activity by promoting pre-mRNA mis-splicing. Humans with the germline biallelic *DCPS*LOF mutations fail to exhibit aberrant hematologic phenotypes, suggesting that DCPS is not critical for human hematopoiesis. These findings provide additional support for a pre-mRNA metabolic pathway that identifies *DCPS* as a potential target for AML therapy [108].

ALL is a common malignancy seen in pediatric patients. It is caused by lymphoid progenitor clonal proliferation in the bone marrow. It represents around 80% of acute leukemia cases in children and only 20% in adult patients. Infiltration of the bone marrow leads to various cytopenias in the peripheral blood associated with the appearance of peripheral blast cells [115].

Swaroop et al., in 2019, using gene-edited cell lines, investigated the impact of mutant NSD2 enzyme in silico, in vitro, and in vivo. In childhood ALL, NSD2, a histone methyltransferase that methylates histone 3 lysine 36 (H3K36), has a glutamic acid to lysine mutation at residue 1099 (E1099K), and cells with this mutation can become the dominant clone in recurrent illness. Cell lines that contained the E1099K mutation had increased H3K36 dimethylation and reduced H3K27 trimethylation, especially in histone H3.1 nucleosomes. These effects were noted to be secondary to altered enzyme/substrate binding and enhanced methylation rate of H3K36 as a result of the E1099K mutation. Mutant NSD2 cells were noted to have reduced apoptosis but increased cell proliferation, adhesion, migration, and clonogenicity. Mutant NSD2 cells had a greater probability of getting through the blood–brain barrier and are more deadly than wild-type cells, according to research using murine xenografts. The use of transcriptional profiling provides evidence that mutant NSD2 stimulates mechanistic pathways associated with signaling and adhesion genes as well as neural and stromal lineages. Understanding the roles of NSD2 and E1099K mutations presents possible targets for future novel therapeutics [116].

Researchers from China reported in 2019, bone marrow transplantation was successfully carried out in a *Human Immunodeficiency Virus-1* (HIV-1)-positive patient suffering from acute lymphocytic leukemia. The patient’s hematopoietic progenitor cells had been *CCR5*-ablated using CRISPR in order to prevent HIV infection [117]. *CCR5* gene encodes for C-C chemokine receptor type 5 (CCR5) protein. *CCR5* ablation was confirmed in the patient’s T cells after engraftment, and off-target effects could be identified by whole-genome sequencing [117,118].

## 3. Perspectives

CRISPR/Cas9 genome editing opens up a world of possibilities for developing next-generation T cell products to tackle cancer and other disorders. Off-target consequences produced by non-specifically detecting unwanted target locations are the key risk for employing CRISPR/Cas9 in human disease treatment. Progress has been made that might considerably reduce off-target effects. As a result, CRISPR/Cas9 technology holds a lot of promise for the future of adoptive cell therapies [119,120,121,122].

The first clinical trials involving CRISPR/Cas9 in humans were initiated in 2016 [78,123]. Researchers isolated immune cells from a patient’s blood and, using CRISPR/Cas9, disabled a gene in the cells. The disabled gene encoding for protein PD-1 usually blocks a cell’s immune response. Some types of cancers take advantage of the PD-1 protein’s function to proliferate. Then they placed the edited cells in cultures, thus increasing their number, and finally administered them to the patient suffering frommetastatic non-small-cell lung cancer. Their hope was that, without functional PD-1 protein, the edited cells would be able to attack and defeat cancer [123].

CRISPRa (CRISPR activation) for gene expression upregulation and CRISPRi (CRISPR interference) for gene expression suppression are two novel CRISPR/Cas9 technologies that have recently been created to modify gene expression [124]. These methods rely on an enzymatically defective Cas9 (dCas9, which is generated by inserting mutations into two nuclease domains) that interacts with or is coupled with transcriptional activators [125]. dCas9 nucleases can still attach to certain DNA sequences, preventing these genes from being transcribed (CRISPRi) [126]. The GAS6-AS2 lncRNA cell line was transcriptionally activated (by CRISPRa), and researchers discovered a hyperactivation of the GAS6/TAM pathway. Multiple malignancies, including AML, have been shown to use this pathway as a resistance strategy [127,128]. CRISPRa has also been explored in bacterial systems, and it appears to be a promising tool for future bacterial engineering [129].

**Figure 2:**
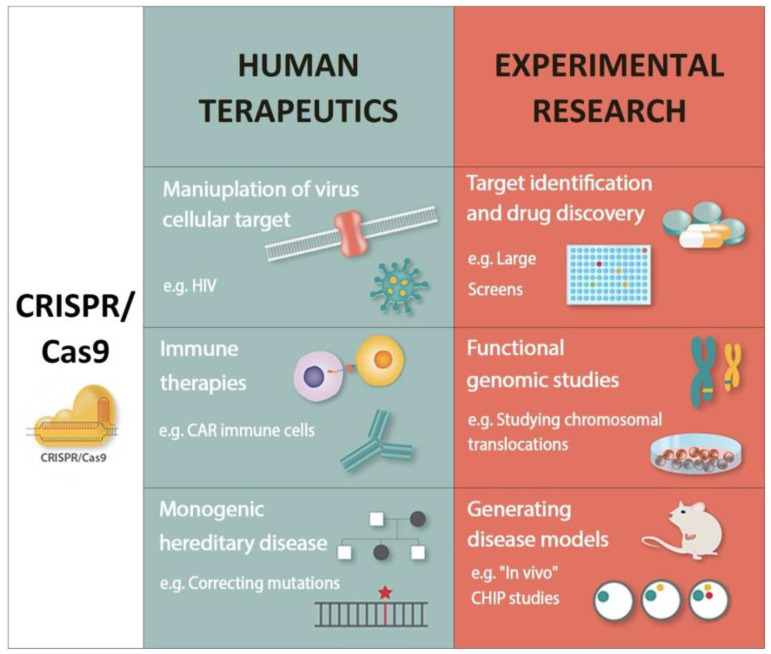
Applications of CRISPR/Cas9 technology in human therapy and hematology research [68]. CRISPR/Cas9 = clustered regularly interspaced short palindromic repeats- associated protein 9, HIV = *Human Immunodeficiency Virus*, CAR = Chimeric antigen receptor, CHIP = clonal hematopoiesis with an indeterminate potential.

CRISPR technology is being used in a variety of ways right now. Figure 2 depicts a quick overview of them.

Several clinical studies investigating the safety and effectiveness of CRISPR-edited cells in the treatment of relapsed or refractory hematological malignancies are underway. NCT04767308 studies CT125A cells in CLL, MCL, DLBCL, FL, and PTCL, where endogenous CD5 in CT125A cells are knocked out by CRISPR/Cas9 to avoid fratricide during CAR-T cell production. CTX110 is being studied in adult B-cell ALL by NCT04035434, while CD19 and CD20 or CD22 CAR-T cells are being studied in B-cell leukemia by NCT03398967. Dual Specificity CD19 and CD20 or CD22 CAR-T cells can detect and destroy CD19-negative malignant cells via CD20 or CD22 identification. PBLTT52CAR19 T cells are being used in one clinical study (NCT04557436) to ensure molecular remission in children with relapsed or refractory B-ALL prior to planned allogeneic stem cell transplantation. For their anti-leukemia properties, gene-edited cells will be employed for a short period of time before being depleted by normal pre-transplant conditioning. XYF19 CAR-T cells with altered endogenous HPK1 (NCT04037566) will be tested in patients with relapsed or refractory CD19+ leukemia or lymphoma in this first-in-human study.

Relapsed or resistant B-cell malignancies can be treated using autologous T cells modified to express CARs against leukemia antigens, such as CD19 on B cells. To address leukemia therapy challenges, researchers are developing universal CD19-specific CAR-T cells called UCAT019. These cells are generated from one or more healthy unrelated donors, which might reduce the risk of graft-versus-host disease (GVHD) as well as lessen their immunogenicity. Allogeneic UCART019 cells with gene-disrupted *TCR* and *B2M* genes have been produced by combining lentiviral delivery of CAR with CRISPR RNA electroporation, and researchers will investigate if it may evade host-mediated immunity and provide anti-leukemic effects without GVHD (NCT03166878). CD7-specific CAR on autologous T cells for cell treatment is another clinical study (NCT03690011).

Basic clinical trial information is summarized in Table 2.

## 4. Challenges and Opportunities for CRISPR/Cas9 Applications in Therapy

### 4.1. Delivering Editing Tools

It is crucial to obtain delivery platforms that can secure the transport of editing components in to various target cells [131]. Ribonucleoprotein is the preferred cargo format because of its “hit-and-run” type mechanism. This method reduces the risk of affecting off-target sites with resultant undesired effects and allows for efficient modification of cells with low translation rates [68]. Less optimal formats include non-integrating viral vectors and mRNA. Taking advantage of the ribonucleoprotein transport format in the CRISPR/Cas9 system further increases the benefit-to-harm ratio. Potential applications include developing novel approaches to integrate donor template DNA and ribonucleoprotein for gene correction in the desired systems [132].

### 4.2. Safety

The most pressing concern regarding the use of CRISPR/Cas9 editing is unintentional Cas9 cleavage leading to off-target DSB [68]. Therefore, it is essential that genome-wide sequencing is applied thoroughly to examine such modifications at unexpected genome sites or at potential off-target sites, which in-silico prediction tools can indicate.

Nevertheless, the consequences of the off-target activity are controversial, and studies have shown discordant results [133,134].

There are several methods to detect CRISPR off-target mutations [135]; however, there is no consensus regarding identifying which potential off-target sites require examination using deep targeted sequencing. Cas9-related on-target mutagenesis, which includes large gene rearrangements and deletions resulting in pathogenic consequences, has been identified as another safety concern [136]. Accordingly, additional studies are needed to better understand the in vivo effects of the CRISPR/Cas9 technique over the human genome. The possibility of undesired gene mutations raises some concerns about the therapeutic utility of the CRISPR/Cas9 system in humans. Infused gene-edited hematopoietic stem cells may have the potential to expand clonally and thereby promote the development of leukemia. One possible solution would be to substitute Cas9 with a different alternative nuclease, such as Cas12a (Cpf1), which prevents mismatches along the 18 nucleotides adjacent to the neighboring protospacer motif [137]. Other solutions could involve paired nickases [138] guided by two unique gRNAs, both targeting the identical locus but on the opposite DNA strands, or “base editors” which edit nucleotides without causing a DNA break [139].

### 4.3. Efficiency

Insufficient target conversion or suboptimal DNA repair outcomes might prevent the desired therapeutic effect of gene editing. Strategies to optimize HDR in CRISPR/Cas9-mediated transgenesis, such as the use of siRNA or shRNA, fusion of Cas9 with a domain of CtIP, or the use of Rad51 activator RS1 that increase the CRISPR/Cas9 efficiency, etc., are reported but require further clinical testing [140]. Efficiency may also be reduced if a mutation from CRISPR/Cas9 is detrimental to cells, resulting in a non-reversible, negative effect [68].

### 4.4. Strategies

Compared to the traditional CRISPR/Cas method, prime editing (PE) has fewer off-target effects and can repair various types of genetic variations (frameshift mutations caused by indels, nucleotide substitutions, etc.) that are linked to human illnesses [141,142]. Two proteins, a Cas9 nickase domain and an engineered reverse transcriptase domain are fused together for PE [143,144,145]. Anzalone AV et al. [141] utilized PE to repair sickle cell anemia’s pathogenic mutation (A > T transversion). For safety concerns, however, more research is necessary, taking into account the indels frequency of PE [146].

A study by Ren J et al. [147,148] used multiplex assays to simultaneously target the two micro globulins, programmed cell death protein 1, and T-cell receptor, etc. This resulted in dual and triple gene ablation with promising results in the generation of CAR-T cells after a single shot. It is required to repair two or more loci at the same time for some illnesses. However, this might lead to off-target consequences, such as chromosomal rearrangement and translocation [145]. There may be a way to circumvent this restriction by using the CRISPR-nickase genome editing technology. CRISPR-nickase, according to Satomura A et al., has no restrictions on editable bases, and off-target effects were not found [149].

### 4.5. Immunogenicity

The immune system’s reaction to genetically modified ex vivo cells or gene editing reagents administered in vivo is also a potential cause for concern [150]. For example, the existence of anti-Cas9 antibodies, mostly isolated from *Staphylococcus pyogenes* or Streptococcus aureus, is common in adults and neonates. Similarly, T lymphocytes targeting *Staphylococcus aureus* Cas9 represent an obstacle to therapeutic gene-editing through CRISPR/Cas9 [151]. Therefore, the role of the immune system as it relates to gene editing must be carefully examined. Strategies to overcome these challenges include incorporating alternative nucleases that have not been exposed to the human immune system or using novel nucleases that lack an immune response. Other strategies include: (1) to recognize the innate immune mechanism that is formed against CRISPR/Cas9 in order to optimize vector choice and engineering; (2) to initiate immunosuppression through medications and/or regulatory T cells to suppress undesired immune reactivity; (3) to construct an in silico prediction tool to assist in determining immunogenic predisposition; and (4) to identify regions on CRISPR/Cas9 that are antigenic with the intention of enabling epitope masking and deimmunization [152].

### 4.6. DNA Damage Response Mediated by TP53

The DNA damage response through *TP53* in some human cell types induced by CRISPR/Cas9 genome editing [153,154] is likely responsible for the low efficiency rates in these cells. Inhibiting *TP53* could theoretically improve the efficiency of genome editing in wild-type cells; however, this may increase the risk of cells being malignantly transformed. Therefore, the sequence and function of the *TP53* gene should be closely monitored in targeted cell populations that are chosen for CRISPR/Cas9 cell-based therapy [68].

### 4.7. Bioethical Regulation

Utilizing CRISPR/Cas9 gene-editing tools is associated with a variety of ethical concerns, including its application to germline cells, embryos, and humans [155,156]. While the clinical application of CRISPR in human somatic cells with the intention of treating hematologic diseases is generally accepted, the consensus among geneticists is that its application in human germline cells and embryos (apart from research purposes), in which future generations would inherit genetic changes, should be impermissible [68]. Recently, it was reported that CRISPR/Cas9 was used to inactivate the CCR5 receptor in human embryos, thereby promoting resistance to HIV infection [157]. Limited and non-peer-reviewed data was presented that described the birth of twin girls that were genetically edited using CRISPR/Cas9. This claim highlights the importance of having clear regulations regarding the use of human CRISPR/Cas9 genome-editing techniques, as its use should be focused on therapeutic applications, not on eugenics or human enhancement. CRISPR/Cas9 has the potential as a research tool to better understand disease pathogenesis and early human development. To date, infusions of ex vivo modified T cells have been given to patients, but none have been treated using in vivo CRISPR-based therapy [68].

The regulatory and ethical considerations involving using CRISPR/Cas9 genome editing for therapeutic purposes are very complex [158]. It is clear that CRISPR/Cas9 has significant potential to modify the human genome, and there are high expectations for future applications. In order to adequately assess concerns and potential benefits, a multidisciplinary regulatory committee involving lawyers, geneticists, clinicians, and society representatives should be established to outline a legislative framework to appropriately regulate the prohibition or permission of CRISPR applications and other genome engineering techniques in the future. Global biological and scientific ethics communities must establish procedures and standards that reduce the risks of these powerful new tools without forgetting the benefits [68].

In conclusion, we can state that CRISPR methods are beneficial for the identification of novel biomarkers for leukemogenesis and disease progression as well as for targeted treatment. A number of techniques have been devised in order to circumvent the real limitations. As a result of medical advances, the overall survival of leukemia patients has improved, although, for some, such as AML, substantial hurdles for long-term life remain. In addition to leukemias, CRISPR-based methods may provide promise for the future of cancer treatment.

## Figures and Tables

**Table 1:** Characteristics of gene editing techniques.

	ZFNs	TALENs	CRISPR/Cas9
Class	Protein DNA	Protein DNA	RNA—DNA
*Zebrafish* genome targeting coverage	Target site every 140–400 base pair	Target site every 1–3 base pair	Target site every 8–128 base pair (NGG PAM)
Technology cost	High	High	Low
Restriction of target site	High G	Start with T, end with A	PAM sequence
Technology adoption time	High	Moderate	Low
Enginereed nucleases	Fok I	Fok I	Cas9
Targeting domain	Zinc-finger domain	Repeat variant diresidue	CRISPR RNA or guide RNA

Table notes. ZFNs = Zinc finger nucleases, TALEN = transcription activator-like effector nucleases, CRISPR/Cas9 = clustered regularly interspaced short palindromic repeats- associated protein 9 [8,24,41,42,43].

**Table 2:** Ongoing clinical trials in leukemia using the CRISPR/Cas9 system [130].

Condition or Disease	Intervention/Treatment	CT Identifier	Phase	First Posted	Title
D5+ Relapsed/Refractory Hematopoietic Malignancies Chronic Lymphocytic Leukemia Mantle Cell Lymphoma Diffuse Large B-cell Lymphoma Follicular Lymphoma Peripheral T-cell Lymphomas	Biological: CT125A cells Drug: Cyclophosphamide, fludarabine	NCT04767308	Early Phase 1	2021	Safety and Efficacy of CT125A Cells for Treatment of Relapsed/Refractory CD5+ Hematopoietic Malignancies
B Acute Lymphoblastic Leukemia	Drug: PBLTT52CAR19	NCT04557436	Phase 1	2020	TT52CAR19 Therapy for B-cell Acute Lymphoblastic Leukemia (PBLTT52CAR19)
B-cell Malignancy Non-Hodgkin Lymphoma B-cell Lymphoma Adult B Cell ALL	Biological: CTX110	NCT04035434	Phase 1	2019	A Safety and Efficacy Study Evaluating CTX110 in Subjects with Relapsed or Refractory B-Cell Malignancies (CARBON)
Acute Lymphocytic Leukemia in Relapse, ALL Refractory Lymphoma, B-Cell CD19 Positive	Genetic: XYF19 CAR-T cell Drug: Cyclophosphamide Drug: Fludarabine	NCT04037566	Phase 1	2019	CRISPR (HPK1) Edited CD19-specific CAR-T Cells (XYF19 CAR-T Cells) for CD19+ Leukemia or Lymphoma
B-cell leukemia B-cell lymphoma	Biological: Universal Dual Specificity CD19 and CD20 or CD22 CAR-T Cells	NCT03398967	Phase 1 Phase 2	2018	A Feasibility and Safety Study of Universal Dual Specificity CD19 and CD20 or CD22 CAR-T Cell Immunotherapy for Relapsed or Refractory Leukemia and Lymphoma
T-cell Acute Lymphoblastic Leukemia T-cell Acute Lymphoblastic Lymphoma T-non-Hodgkin Lymphoma	Genetic: CD7.CAR/28zeta CAR T cells Drug: Fludarabine Drug: Cytoxan	NCT03690011	Phase 1	2018	Cell Therapy for High Risk T-Cell Malignancies Using CD7-Specific CAR Expressed On Autologous T Cells
B-cell leukemia B-cell lymphoma	Biological: UCART019	NCT03166878	Phase 1 Phase 2	2017	A Study Evaluating UCART019 in Patients with Relapsed or Refractory CD19+ Leukemia and Lymphoma

CAR T cells = Chimeric antigen receptor T cells.

## Data Availability

Not applicable.
